# Revolutionizing Healthcare: The Emerging Role of Quantum Computing in Enhancing Medical Technology and Treatment

**DOI:** 10.7759/cureus.67486

**Published:** 2024-08-22

**Authors:** Naveen Jeyaraman, Madhan Jeyaraman, Sankalp Yadav, Swaminathan Ramasubramanian, Sangeetha Balaji

**Affiliations:** 1 Orthopaedics, ACS Medical College and Hospital, Dr MGR Educational and Research Institute, Chennai, IND; 2 Orthopaedics, South Texas Orthopaedic Research Institute, Texas, USA; 3 Clinical Research Associate, Viriginia Tech India, Dr MGR Educational and Research Institute, Chennai, IND; 4 Medicine, Shri Madan Lal Khurana Chest Clinic, New Delhi, IND; 5 Orthopaedics, Government Medical College, Omandurar Government Estate, Chennai, IND

**Keywords:** personalized medicine, drug discovery, data processing, diagnostics, healthcare, quantum computing

## Abstract

The healthcare sector faces complex challenges that call for innovative solutions to improve diagnostic accuracy, treatment efficacy, and data management. Quantum computing, with its unique capabilities, holds the potential to revolutionize various aspects of healthcare. This narrative review critically examines the existing literature on the application of quantum computing in healthcare, focusing on its utility in enhancing diagnostics, data processing, and treatment planning. Quantum computing's ability to handle large, complex datasets more efficiently than classical computers can significantly impact domains such as genomics, medical imaging, and personalized medicine. Quantum algorithms can accelerate the identification of genetic markers associated with diseases, facilitate the analysis of medical images, and optimize treatment plans based on individual genetic profiles. Moreover, quantum cryptography offers a robust security solution for safeguarding sensitive patient data, a critical need as healthcare increasingly relies on digital platforms. Despite the promising outlook, the integration of quantum computing into healthcare faces technical, ethical, and regulatory challenges. The delicate nature of quantum hardware, the need for error correction, and the scalability of quantum systems pose barriers to widespread adoption. Additionally, concerns around patient privacy and data security, as well as the need for updated regulatory frameworks, must be addressed. Ongoing research and collaborative efforts involving researchers, healthcare providers, and technology developers are crucial to overcoming these hurdles and realizing the full potential of quantum computing in transforming healthcare. As quantum computing continues to evolve, its impact on the future of healthcare could be profound, leading to earlier disease detection, more personalized treatments, and improved patient outcomes. For instance, quantum computing has already been applied to enhance drug discovery processes, with companies like D-Wave Systems (Burnaby, Canada) demonstrating faster molecular simulations for pharmaceutical research and IBM's (Armonk, USA) quantum systems being used to model chemical reactions for new drug development.

## Introduction and background

**Figure 1:**
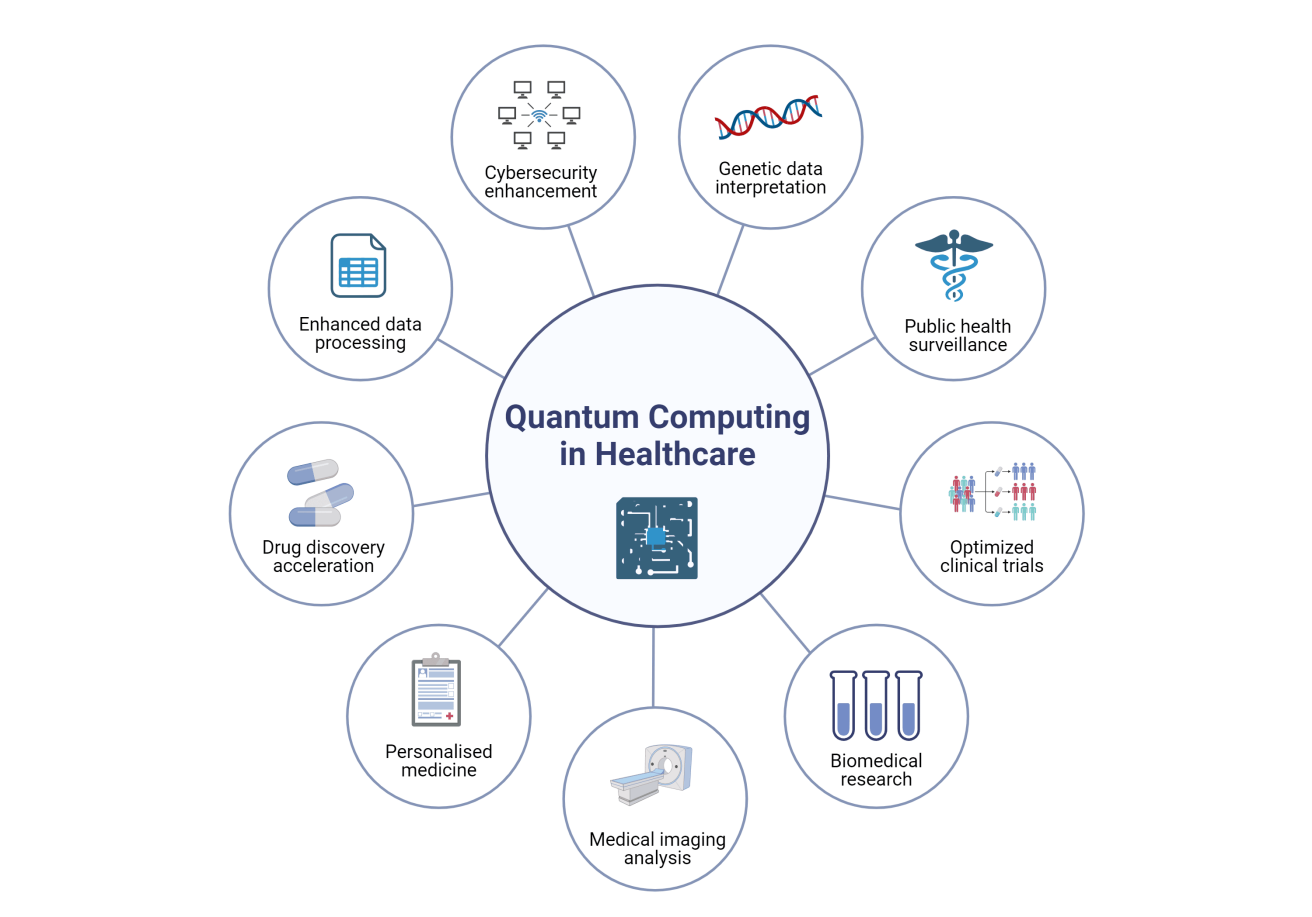
Domains of quantum computing in the healthcare industry Picture courtesy: Dr. Naveen Jeyaraman

The healthcare sector is increasingly facing complex challenges that stretch across diagnostic accuracy, treatment efficacy, data management, and patient-specific therapy planning. Traditional computational methods are often limited by their linear scaling and processing capabilities, which restricts their ability to manage and analyze the exponentially growing datasets in healthcare. Quantum computing, heralded for its potential to process complex datasets far more efficiently than classical computers, could be a pivotal solution to these issues [1,2]. For instance, quantum computing could enhance biomarker discovery by analyzing vast datasets to identify subtle patterns that are indiscernible to classical methods [3]. Furthermore, in diagnostics, quantum computing applications show promising advances in imaging techniques and early disease detection by improving the speed and accuracy of the analyses required to interpret medical images and genetic information [3-7]. The potential of quantum computing in healthcare is vast and varied (Figure 1).

One of the primary advantages is its ability to revolutionize data processing through quantum parallelism, which allows for the simultaneous processing of information, drastically reducing the time required for complex computations such as molecular simulations in drug discovery [8]. This capability could dramatically shorten development timelines and improve the precision of therapeutic interventions. Additionally, in diagnostics, quantum-enhanced algorithms could improve the sensitivity and specificity of diagnostic tools, allowing for earlier detection of conditions like cancer through enhanced imaging technologies and more accurate interpretation of radiographic data [9]. Moreover, quantum computing can contribute significantly to personalized medicine by optimizing treatment plans based on individual genetic profiles, thus enhancing the efficacy and minimizing the side effects of treatments [3,10].

The goal of this narrative review is to critically examine the existing literature on the application of quantum computing in healthcare, with a specific focus on its utility in improving diagnostics, data processing, and treatment planning. This review aims to synthesize current findings, highlight critical advancements, and discuss the practical implications of integrating quantum technologies into medical practice. By doing so, it seeks to provide a comprehensive overview of how quantum computing could address the pressing challenges in today’s healthcare landscape, thereby setting the stage for future research and implementation.

## Review

Quantum computing in healthcare

Quantum computing represents a fundamental shift from classical computing paradigms, leveraging the principles of quantum mechanics to process information in profoundly different ways. At the core of quantum computing are qubits, which, unlike classical bits that represent data as 0s or 1s, can exist simultaneously in multiple states due to superposition [3]. This allows quantum computers to perform many calculations at once, enhancing their computing power exponentially as more qubits are entangled. Entanglement, another cornerstone of quantum mechanics, involves linking qubits in such a way that the state of one (whether it's in position, momentum, spin, etc.) directly correlates with the state of another, regardless of the distance separating them [10]. The potential of quantum algorithms is immense. For instance, algorithms like Shor's, which is used for factoring large numbers, and Grover's, which enhances database search capabilities, could dramatically speed up tasks ranging from securing patient data with advanced cryptographic measures to searching large medical databases more efficiently [3,8]. This property is crucial for quantum algorithms, which can solve problems more efficiently than their classical counterparts, particularly in fields requiring complex pattern recognition and data-intensive simulations, such as biomolecular modeling in drug discovery [8,11].

The healthcare industry currently employs various advanced technologies, including electronic health records (EHRs), diagnostic imaging tools like MRI and CT scanners, and complex bioinformatics systems for genomics. However, these technologies face significant challenges, including the management of large data volumes and the need for high-speed processing and accurate analysis. For instance, EHR systems often struggle with interoperability issues, which hinder the effective sharing of patient data across different healthcare platforms [9]. Similarly, while imaging technologies like MRI and CT scans provide valuable diagnostic information, they require substantial computational resources to process and analyze the data they produce. These limitations are pronounced in the handling of genetic information, where the vast amount of data generated by sequencing technologies demands extensive computational power to derive meaningful insights [12]. Quantum computing has the potential to overcome these limitations by enabling faster and more precise computations. For example, quantum algorithms could enhance the analysis of imaging data, reducing the time required for diagnosing diseases and potentially improving the accuracy of these diagnoses [9,12]. Moreover, in the field of genomics, quantum computing could expedite the process of sifting through massive datasets to identify genetic markers linked to specific diseases, thus speeding up the diagnosis and personalization of treatment plans [3,10]. While still in the early stages, real-world implementations of quantum computing in healthcare are emerging. For example, the Cleveland Clinic has partnered with IBM to establish the Discovery Accelerator, which uses quantum computing for drug discovery and personalized medicine research. Additionally, Zapata Computing has collaborated with pharmaceutical companies to optimize clinical trial design using quantum-inspired algorithms.

Extensive research has been conducted to explore the integration of quantum computing into various aspects of healthcare. For example, studies have examined how quantum computing can enhance drug discovery by simulating molecular interactions, which could potentially reduce the time and cost associated with developing new drugs [8]. Other research has focused on the application of quantum principles to improve diagnostic processes, such as enhancing the capabilities of imaging technologies through faster and more accurate data processing [9,13]. Several reviews have also highlighted the potential of quantum computing to revolutionize personalized medicine [14,15]. By leveraging quantum algorithms, healthcare providers could tailor treatments to individual patients based on their unique genetic profiles, thereby improving treatment efficacy and reducing side effects [10]. Furthermore, quantum computing could aid in the development of new diagnostic tools and techniques, offering greater precision in identifying and treating various medical conditions [3].

Data security and privacy

The integration of quantum computing into healthcare promises to revolutionize the way sensitive medical information is secured and managed. Quantum cryptography, in particular, offers an advanced layer of security that is based on the principles of quantum mechanics rather than traditional mathematical algorithms, making it potentially impervious to all forms of cyberattack [16]. Quantum cryptography utilizes the principles of quantum mechanics, specifically the behavior of quantum bits, or qubits, to establish secure communication channels [17]. The most notable quantum cryptographic technique is quantum key distribution (QKD), which allows two parties to generate a shared random secret key known only to them, which can then be used to encrypt and decrypt messages [18]. An inherent property of quantum mechanics is that measuring a quantum system inevitably disturbs it. Therefore, any eavesdropping attempt on QKD transmissions can be detected immediately, as it introduces detectable anomalies in the communication [9,12]. In healthcare, where the confidentiality and integrity of patient data are paramount, quantum cryptography can secure the transmission of electronic health records (EHRs) between different healthcare entities. As healthcare systems increasingly adopt digital solutions, the risk of cyberattacks grows. Quantum cryptography provides a solution that could potentially safeguard data against even the most sophisticated cyber threats, ensuring that patient data remains private and tamper-proof. This application is critically discussed by Shams et al. and Tarasov et al., which outline the vulnerabilities in current EHR systems and the potential for quantum technologies to mitigate these risks [3,19]. Despite these challenges, several pilot projects are underway. For instance, the Dutch bank ABN AMRO, in collaboration with QuTech, has successfully tested a quantum key distribution network for securing financial transactions, a model that could be adapted for healthcare data protection. In the UK, BT and Toshiba have implemented a quantum-secured network between the National Composites Centre and the Centre for Modelling and Simulation, demonstrating the potential for securing sensitive healthcare data transfers.

The rise of telemedicine, especially highlighted during global health crises like the COVID-19 pandemic, has necessitated robust security solutions for remote healthcare services. Quantum cryptography can play a vital role in protecting data exchanged during telehealth sessions, which often include sensitive health information and personal data [20,21]. Implementing quantum-secured communication channels for telehealth can prevent unauthorized access and ensure the confidentiality and integrity of remote consultations [10,22]. Despite its potential, the integration of quantum cryptography into healthcare IT systems faces several challenges. The technology requires significant infrastructure investments and is currently more expensive than traditional cryptographic methods. Additionally, the healthcare sector must overcome various technical and regulatory hurdles to adopt this new technology, including updating existing systems and ensuring compliance with health data protection laws [5,9]. There are ongoing pilot projects and theoretical models testing the application of quantum cryptography in healthcare settings. For example, projects discussed by Dhande, Bagachi, and Arshad et al. involve a quantum-secured network connecting several hospitals and healthcare providers, designed to protect patient data as it moves across different systems and stakeholders [8,23]. Studies by Solenov and Rasool et al. examine the feasibility of using quantum cryptography for securing wearable health monitoring devices, which collect and transmit vast amounts of health data in real time [24,15]. Another promising area of research is the integration of quantum cryptography with blockchain technology, which could further enhance the security of healthcare systems. Blockchain's decentralized nature, combined with quantum cryptography's unbreakable security, could create a nearly impenetrable system for managing health records and transactions, preventing fraud and unauthorized access [8].

Big data analytics in healthcare

The explosion of data within the healthcare sector, especially from genomic sequencing and electronic health records, poses unique challenges in data management and analysis. Quantum computing offers transformative solutions for big data analytics in healthcare by leveraging its inherent ability to handle vast datasets more efficiently than classical computers [25-27]. Quantum algorithms are particularly suited for the analysis of genetic information due to their ability to perform parallel computations and manage large-scale combinatorial problems, which are common in genomics. The ability to quickly sift through massive genomic datasets can significantly enhance the identification of genetic markers associated with diseases, improving diagnostic accuracy and treatment outcomes. Padalhin et al. and Tarasov et al. explore how quantum computing can accelerate the processing of genomic data, facilitating more rapid and precise genetic analysis compared to classical methods [3,10]. This is particularly crucial in the era of personalized medicine, where understanding a patient’s genetic profile is essential for tailoring specific therapeutic strategies.

In epidemiology, the analysis of large datasets to track disease patterns, outbreaks, and the effectiveness of interventions requires substantial computational resources. Quantum computers, with their superior processing capabilities, could dramatically improve the speed and accuracy of data analysis in epidemiological studies. For instance, Flöther discusses how quantum algorithms could be used to model complex interactions within large populations quickly, thereby enhancing the ability to predict and manage public health crises [5]. Similarly, Giang highlights the application of quantum computing in real-time disease surveillance and response systems, which can benefit from the quick processing of data from various sources, including healthcare facilities and social media [12]. The general capability of quantum computers to handle large datasets can be applied to various other aspects of healthcare beyond genomics and epidemiology. Dhande and Bagchi detailed how quantum computing could transform the analysis of patient data from continuous monitoring devices and mobile health applications [8]. These devices generate vast amounts of data that, when analyzed effectively, can provide insights into patient health trends, disease progression, and the effectiveness of treatments. Quantum algorithms can enhance the analysis of this data, enabling healthcare providers to make more informed decisions faster.

Practical implementations and theoretical models provide a glimpse into how quantum computing could reshape big data analytics in healthcare. For instance, the studies by Arshad et al. and Solenov et al. examine the use of quantum machine learning algorithms to predict patient outcomes based on historical health data, showcasing a potential reduction in the time required for data processing and analysis [23,24]. Another example, as seen in the article by Maheshwari et al., involves the use of quantum algorithms to enhance the precision of imaging techniques, which are data-intensive and require significant computational power to analyze [22]. Integrating quantum computing with existing healthcare data systems poses technical challenges but offers substantial rewards. By augmenting classical computing systems with quantum processors for specific tasks, healthcare organizations can achieve a balance of efficiency and practicality. This hybrid approach allows for the gradual integration of quantum capabilities into healthcare analytics, enhancing existing infrastructures rather than replacing them outright [15]. Practical implementations are beginning to emerge. For example, Accenture and 1QBit have developed a quantum-enabled molecular comparison application that could significantly speed up drug discovery processes. Meanwhile, QC Ware and Merck have partnered to develop quantum algorithms for chemical simulations, demonstrating the potential for more efficient pharmaceutical research and development.

Drug discovery

Quantum computing holds transformative potential for the field of drug discovery, offering unprecedented capabilities in simulating molecular interactions and accelerating the design of new pharmaceuticals [11,28,29]. The enhanced computational power of quantum computers can drastically reduce the time and resources required for drug development, from initial screening to final testing. One of the primary applications of quantum computing in drug discovery is the simulation of molecular interactions. Classical computers often struggle with the complexity and scale of molecular simulations due to the exponential growth of variables with each additional atom in the system. Quantum computers, however, can handle these complexities more efficiently. For instance, Dhande, Bagchi, and Tarasov et al. discuss how quantum algorithms enable the simulation of biochemical processes that would be infeasible with classical computing [3,8]. These capabilities allow for a more accurate prediction of how drug molecules interact with biological targets, which is crucial for designing effective drugs. Quantum computing can also revolutionize the screening process in drug discovery. Traditional methods for drug screening involve testing thousands of compounds, which can be time-consuming and costly. Quantum algorithms improve the efficiency of this process by enabling the rapid identification of promising compounds based on their interaction properties. Quantum computing is used to analyze large libraries of compounds, significantly speeding up the discovery of potential new drugs [30].

Several case studies underscore the practical applications of quantum computing in drug discovery. For example, Padalhin et al. and Maheshwari et al. describe how quantum computing has been applied to optimize the molecular structure of drugs, enhancing their efficacy and reducing side effects [10,22]. These studies show that quantum computing not only speeds up the process but also improves the quality of the outcomes, leading to more effective and safer medications [31,32]. Theoretical models of quantum computing in drug discovery provide a framework for understanding the potential impact of this technology. These models suggest that quantum computing could reduce the drug development timeline by several years, translating into significant cost savings for pharmaceutical companies and faster access to new treatments for patients. Arshad et al. and Solenov et al. explore these models, illustrating how quantum-enhanced algorithms could lead to breakthroughs in treating complex diseases like cancer and Alzheimer's by uncovering novel therapeutic targets [23,24]. Beyond the initial phases of drug discovery, quantum computing also plays a role in predicting the outcomes of drug interactions. This capability is vital for assessing drug safety and efficacy before clinical trials. Flöther discusses how quantum simulations can predict the pharmacokinetics and pharmacodynamics of new drugs, providing insights into their behavior in the human body, which is essential for ensuring patient safety [5]. Real-world applications are already underway. For instance, Google's quantum computer has successfully simulated a simple chemical reaction, laying the groundwork for more complex pharmaceutical simulations. Additionally, Polaris Quantum Biotech has used quantum computing to identify potential drug candidates for COVID-19, demonstrating the technology's potential in rapid response to health crises. However, implementing quantum computing in drug discovery faces practical challenges. The current limitation in qubit coherence time restricts the complexity of molecular simulations. To address this, researchers are developing hybrid quantum-classical algorithms, such as the Variational Quantum Eigensolver (VQE), which leverages both quantum and classical resources to mitigate the effects of noise and errors. Additionally, the need for domain-specific expertise in both quantum computing and pharmaceutical sciences presents a workforce challenge. Companies like Zapata Computing are addressing this by offering specialized training programs that bridge the knowledge gap between quantum physics and drug discovery processes.

Personalized medicine

Quantum computing's role in personalized medicine is an exciting frontier, promising to tailor medical treatments to individual patients based on their unique genetic profiles. This personalized approach is crucial for optimizing therapeutic efficacy and minimizing adverse effects, thereby revolutionizing patient care. Personalized medicine relies heavily on the ability to analyze and interpret large volumes of genetic data to determine the most effective treatment for each individual. Quantum computing enhances this process through its superior computational power, which allows for the rapid analysis of genetic information. For instance, Padalhin et al. and Tarasov et al. discuss how quantum algorithms can be employed to decipher complex genetic markers and interactions that determine a patient’s response to various medications [3,10]. This capability enables clinicians to prescribe drugs that are most likely to be effective based on a patient's genetic makeup. Quantum computing also contributes to personalized medicine by improving diagnostic accuracy. With quantum-enhanced imaging techniques and data analysis, doctors can detect diseases at earlier stages and with greater precision. Shan et al. highlight how quantum computing facilitates the development of advanced diagnostic tools that integrate genetic, environmental, and lifestyle factors, providing a more comprehensive assessment of a patient's health and disease risk [33].

Practical applications of quantum computing in personalized medicine are already being explored. Shams et al. and Maheswari et al. provide examples of how quantum-driven models have improved the precision of cancer therapies by predicting how cancer cells will react to different treatments based on genetic variations [19,22]. These studies illustrate the potential for quantum computing to significantly enhance treatment outcomes by adapting therapies to the individual characteristics of each patient’s disease. The impact of quantum computing on treatment outcomes and efficiency in personalized medicine is profound. By enabling more precise and predictive medicine, quantum technology helps reduce the trial-and-error approach often associated with treatment planning [34,35]. This not only improves patient outcomes but also reduces healthcare costs associated with ineffective treatments. Giang and Almotiri et al. detail how quantum algorithms streamline the process of treatment design, making healthcare delivery more efficient and effective [9,12]. Another important aspect of personalized medicine is the management of drug interactions, particularly in patients with complex medical histories. Quantum computing aids in modeling and predicting the interactions between various medications, which is critical for avoiding adverse drug reactions. Solenov et al. and Flöther discuss how quantum simulations provide insights into the molecular basis of drug interactions, offering a more detailed understanding that can guide the development of safer medication regimes tailored to individual patients [5,24]. While quantum computing shows promise in personalized medicine, significant challenges remain. The current state of quantum hardware limits the scale of genomic data that can be processed effectively. Researchers at the University of Virginia are addressing this by developing quantum-inspired algorithms that can run on classical computers, providing a bridge to full quantum implementations. Another challenge is integrating quantum-derived insights into existing clinical workflows. To tackle this, IBM and the Cleveland Clinic are collaborating on a quantum computing platform that aims to seamlessly incorporate genomic analysis into clinical decision support systems.

Quantum computing for healthcare research

Quantum computing is poised to revolutionize healthcare research by offering new ways to solve complex problems that are intractable for classical computers. This emerging technology can significantly accelerate biomedical research, from understanding disease mechanisms to improving public health surveillance systems [36,37]. At the heart of biomedical research is the need to understand complex biological processes that involve vast amounts of data. Quantum computing can dramatically reduce the time required for computational tasks such as protein folding, genomic sequencing, and molecular dynamics simulations. For instance, Dhande and Bagchi [8] and Shan et al. [33] describe how quantum algorithms are used to simulate the molecular interactions of proteins, providing insights into their structure and function that are critical for drug design and disease understanding. These simulations, which would take years for classical computers to process, can be completed in significantly less time using quantum computers, thereby speeding up the research process and enabling more rapid advancements in medicine. Quantum computing also holds the potential to transform genomic research by enabling the analysis of large genomic datasets more efficiently. The ability to quickly process and analyze vast amounts of genetic data can lead to breakthroughs in understanding the genetic bases of diseases and in identifying new therapeutic targets. For example, Tarasov et al. [3] and Padalhin et al. [10] highlight the use of quantum algorithms in analyzing genetic mutations and their associations with specific diseases, allowing researchers to develop more targeted and effective therapies.

Quantum computing can improve the design and analysis of clinical trials [38-42]. By simulating patient populations and potential outcomes, researchers can optimize trial designs to be more effective and efficient. This includes the ability to predict which patient groups are most likely to benefit from a particular treatment, thus enhancing the precision and reducing the cost of clinical trials. Giang and Almotiri et al. discuss the role of quantum computing in modeling complex interactions within clinical trials, including patient response to treatments and potential side effects, thereby improving the safety and efficacy of new therapies [9,12]. In public health, quantum computing can enhance disease surveillance and control strategies [43-47]. Quantum algorithms can process large-scale epidemiological data to identify patterns and predict outbreaks, facilitating more effective public health interventions. Shams et al. and Flöther explore how quantum computing assists in real-time monitoring and simulation of disease spread, allowing health authorities to make informed decisions quickly during public health emergencies [5,19].

**Figure 2:**
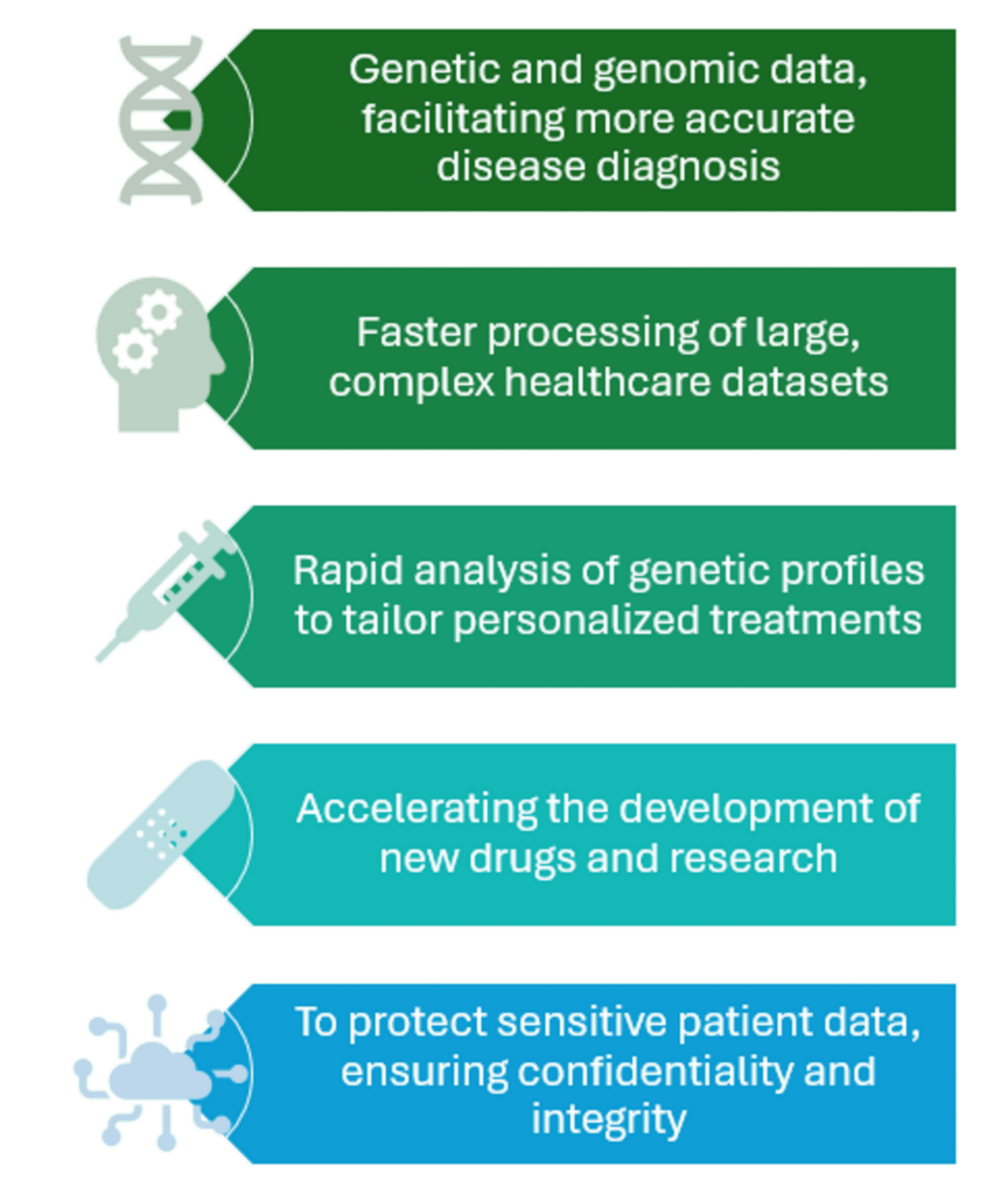
Dimensions of quantum computing in healthcare Picture courtesy: Dr. Sankalp Yadav

Several practical applications and case studies have demonstrated the potential of quantum computing in healthcare research. For instance, Maheshwari et al. outline how quantum machine learning algorithms have been applied to predict hospital readmission rates, a key factor in hospital management and policymaking [22]. Similarly, Arshad et al. [23] and Solenov et al. [24] provide examples of how quantum-driven insights have helped in the early detection and management of cardiovascular diseases by analyzing patient data collected from wearable devices. Advanced imaging techniques are crucial for diagnosing and understanding various medical conditions. Quantum computing enhances these techniques by improving the algorithms used for image reconstruction and analysis, which can lead to better diagnosis and treatment planning. Rasool et al. highlights how quantum algorithms optimize the processing of medical images, such as MRIs and CT scans, making them faster and more accurate, thus providing clinicians with clearer and more detailed images for better patient assessment [15]. The dimensions of quantum computing in healthcare are depicted in Figure 2.

Several cutting-edge research projects are currently leveraging quantum computing in healthcare. For instance, the QuPharma project, a collaboration between the University of Strathclyde and pharmaceutical companies, is using quantum computing to model complex molecular interactions for drug development. In another example, researchers at the University of Sussex are applying quantum algorithms to analyze large-scale brain imaging data, aiming to improve our understanding of neurological disorders. These projects demonstrate the growing integration of quantum computing across various domains of healthcare research.

Challenges and limitations

While quantum computing holds immense potential for revolutionizing healthcare through its superior computational capabilities, its integration into the healthcare sector is not without significant challenges. These challenges range from technical limitations to ethical and regulatory concerns that must be addressed to fully harness quantum technologies in medical settings. One of the foremost technical challenges in the deployment of quantum computing in healthcare is the nascent state of quantum hardware. Quantum computers require extremely delicate conditions to function, such as ultra-low temperatures and environments that are almost entirely free of magnetic and electrical interference. These requirements make quantum computers both expensive and complex to maintain. Moreover, the current generation of quantum computers, often referred to as NISQ (Noisy Intermediate-Scale Quantum) devices, is prone to high error rates due to quantum decoherence and noise, which significantly limit their reliability and scalability for practical applications [5,33].

**Figure 3:**
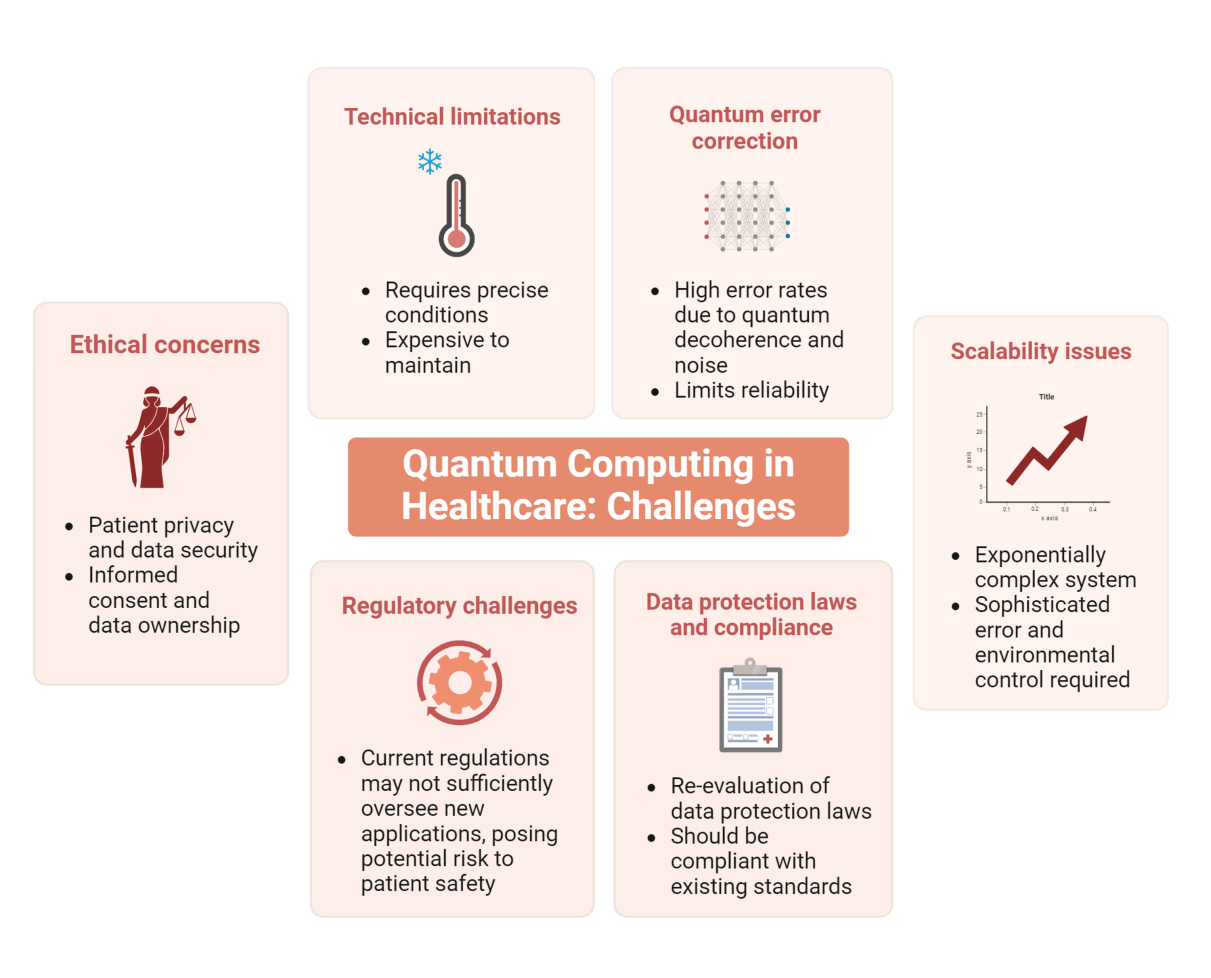
Challenges of quantum computing in healthcare Picture courtesy: Dr. Madhan Jeyaraman

Quantum bits, or qubits, are highly susceptible to errors from even minimal environmental interactions. Quantum error correction is a major area of research, but it requires a substantial overhead of additional qubits to correct the errors of a single qubit, which complicates the scaling of quantum systems. Effective error correction mechanisms are essential for quantum computers to perform complex calculations reliably, especially in applications like drug discovery and genetic data analysis, where accuracy is paramount [3,12]. The scalability of quantum systems is another significant challenge. Increasing the number of qubits is not merely a matter of adding more processors, as in classical computing. Each additional qubit increases the complexity of the system exponentially, requiring even more sophisticated error correction and environmental controls. This makes scaling quantum computers a daunting technical challenge that must be overcome to realize their potential in widespread healthcare applications [10,19]. The challenges of quantum computing in healthcare are depicted in Figure 3.

The application of quantum computing in healthcare raises several ethical concerns, particularly regarding patient privacy and the security of sensitive medical data. The immense power of quantum computers could potentially be used to break traditional encryption methods, leading to concerns about the misuse of patient data. Additionally, the ability of quantum computers to quickly process vast amounts of data raises questions about informed consent and data ownership-issues that are already contentious in the realm of big data and personalized medicine [8,15]. The regulatory frameworks currently in place may not be sufficient to oversee the novel applications and capabilities of quantum technologies in healthcare. The rapid development of quantum computing capabilities could outpace existing regulations, leading to gaps in oversight and potential risks to patient safety. Healthcare regulators will need to develop new guidelines to ensure that quantum computing technologies are implemented safely and ethically. This includes rigorous testing for safety and efficacy, as well as clear guidelines on the management and use of data processed by quantum systems [22,23]. As quantum computing has the potential to revolutionize data security through quantum cryptography, it also necessitates a re-evaluation of data protection laws. Current regulations may not adequately address the security capabilities and challenges posed by quantum technology. Ensuring compliance with data protection standards such as GDPR in Europe and HIPAA in the United States will be crucial as healthcare systems begin to incorporate more advanced quantum computing solutions [8,24]. To address these challenges, several initiatives are underway. The European Quantum Flagship Program includes a focus on quantum computing ethics, working to develop guidelines for responsible quantum technology use in healthcare. In the U.S., the National Quantum Initiative Act has established a subcommittee on quantum information science to address regulatory challenges. On the industry side, IBM's Quantum Safe cryptography project is developing new encryption methods to protect healthcare data in the quantum era. These efforts represent initial steps towards creating a comprehensive framework for ethical and secure quantum computing in healthcare.

Future directions

As quantum computing continues to evolve, its potential applications within the healthcare sector are expanding, driven by innovative research and interdisciplinary collaborations. The future of quantum computing in healthcare looks promising, with several trends and collaborative efforts poised to catalyze significant breakthroughs. One of the most promising trends is the development of hybrid systems that combine quantum and classical computing elements. These systems leverage the strengths of both technologies, using quantum computing for tasks that involve massive parallelism and complex calculations while relying on classical systems for more routine data processing tasks. This approach not only enhances computational efficiency but also provides a practical pathway for integrating quantum computing into existing healthcare IT infrastructures without complete overhauls. Projects like those discussed by Shams et al. and Shan et al. illustrate how hybrid systems are already being tested in fields such as drug discovery and genomic data analysis, showing significant improvements in processing times and accuracy [19,33]. Quantum machine learning (QML) is emerging as a powerful tool for enhancing diagnostic imaging techniques. Quantum algorithms are particularly suited for pattern recognition, which can be applied to complex medical images to identify subtle features that may not be visible to classical algorithms. Ongoing research in developing QML models is focused on improving the accuracy and speed of medical diagnoses from imaging data, with potential applications in early disease detection and automated diagnostic systems [3,22].

The complexity and novelty of quantum computing applications in healthcare necessitate collaborations across several disciplines. Building teams that include quantum physicists, healthcare professionals, bioinformatics experts, and computer scientists can foster a comprehensive approach to tackling the challenges at the intersection of quantum computing and medicine. Such collaborations can drive innovation by combining expertise in quantum mechanics with insights from medical practice and biomedical research, leading to more effective solutions tailored to specific healthcare needs. Collaborations between academic institutions and healthcare industries are crucial for advancing the practical applications of quantum computing in medicine. These partnerships can facilitate the exchange of knowledge, resources, and technologies, accelerating the pace of research and development. For instance, initiatives like those highlighted by Dhande, Bagchi, and Solenov et al. involve collaborations between universities and pharmaceutical companies to develop quantum-based methods for drug discovery and patient data analysis, providing a model for how such partnerships can be structured [8,24]. On a broader scale, there is a growing recognition of the potential for quantum computing to address global health challenges. Collaborative international efforts can leverage quantum computing to improve public health surveillance, manage outbreaks, and enhance global disease prevention strategies. By sharing quantum computing resources and expertise, countries can better prepare for and respond to health crises [10,12]. Concrete examples of such collaborations are emerging. The Quantum Economic Development Consortium (QED-C) in the U.S. brings together industry, academic, and government partners to advance quantum technology applications, including in healthcare. Their ongoing project on quantum sensing for medical imaging aims to develop ultra-sensitive quantum sensors for early disease detection. Internationally, the EU-funded AQTION project unites nine European research institutions to build a quantum computer for pharmaceutical applications. These collaborations are expected to accelerate the development of practical quantum solutions for healthcare challenges, potentially leading to breakthroughs in drug discovery and personalized medicine within the next decade.

## Conclusions

The integration of quantum computing into healthcare offers significant potential to revolutionize the field, particularly in enhancing data processing, improving diagnostic accuracy, optimizing drug discovery, and enabling personalized medicine. These advancements could transform healthcare delivery, leading to better patient outcomes and more efficient resource utilization. However, the current limitations of quantum hardware, including the need for specialized environments and susceptibility to errors, present significant barriers to widespread adoption. Addressing these challenges through continued research and development is crucial, as is careful consideration of the ethical and regulatory implications, especially regarding data privacy and security. To facilitate the integration of quantum computing into healthcare in the near term, a strategic roadmap is essential. Key steps include prioritizing education and training to build a quantum-literate workforce, with healthcare institutions collaborating with academic organizations to develop interdisciplinary curricula that bridge quantum physics and healthcare. Establishing quantum-classical hybrid systems can provide a practical pathway to leverage quantum advantages while operating within current technological limitations. Additionally, healthcare organizations should initiate pilot projects focusing on specific applications, such as drug discovery or personalized medicine, by partnering with quantum technology providers to test and refine these implementations. Regulatory bodies must also proactively develop frameworks to evaluate and approve quantum-based healthcare solutions, ensuring patient safety and data security. Furthermore, fostering open collaboration platforms where researchers, clinicians, and quantum experts can share insights and best practices will be vital to accelerating progress. As quantum computing continues to evolve, its impact on healthcare will depend on overcoming technical, ethical, and regulatory hurdles. With sustained investment and interdisciplinary cooperation, quantum computing has the potential to radically transform the healthcare landscape, enabling earlier disease detection, more personalized treatments, and ultimately improved patient outcomes. Although challenges lie ahead, the benefits that quantum computing can bestow on healthcare make this endeavor a journey worth pursuing.
